# Cerebral metabolism following traumatic brain injury: new discoveries with implications for treatment

**DOI:** 10.3389/fnins.2014.00408

**Published:** 2015-02-09

**Authors:** George A. Brooks, Neil A. Martin

**Affiliations:** ^1^Exercise Physiology Laboratory, Department of Integrative Biology, University of California, BerkeleyBerkeley, CA, USA; ^2^Department of Neurosurgery, David Geffen School of Medicine, University of California, Los AngelesLos Angeles, CA, USA

**Keywords:** lactate shuttle, gluconeogenesis, trauma, brain fuel, brain metabolism

## Abstract

Because it is the product of glycolysis and main substrate for mitochondrial respiration, lactate is the central metabolic intermediate in cerebral energy substrate delivery. Our recent studies on healthy controls and patients following traumatic brain injury (TBI) using [6,6-^2^H_2_]glucose and [3-^13^C]lactate, along with cerebral blood flow (CBF) and arterial-venous (jugular bulb) difference measurements for oxygen, metabolite levels, isotopic enrichments and ^13^CO_2_ show a massive and previously unrecognized mobilization of lactate from corporeal (muscle, skin, and other) glycogen reserves in TBI patients who were studied 5.7 ± 2.2 days after injury at which time brain oxygen consumption and glucose uptake (CMRO_2_ and CMRgluc, respectively) were depressed. By tracking the incorporation of the ^13^C from lactate tracer we found that gluconeogenesis (GNG) from lactate accounted for 67.1 ± 6.9%, of whole-body glucose appearance rate (Ra) in TBI, which was compared to 15.2 ± 2.8% (mean ± SD, respectively) in healthy, well-nourished controls. Standard of care treatment of TBI patients in state-of-the-art facilities by talented and dedicated heath care professionals reveals presence of a catabolic Body Energy State (BES). Results are interpreted to mean that additional nutritive support is required to fuel the body and brain following TBI. Use of a diagnostic to monitor BES to provide health care professionals with actionable data in providing nutritive formulations to fuel the body and brain and achieve exquisite glycemic control are discussed. In particular, the advantages of using inorganic and organic lactate salts, esters and other compounds are examined. To date, several investigations on brain-injured patients with intact hepatic and renal functions show that compared to dextrose + insulin treatment, exogenous lactate infusion results in normal glycemia.

## Glycolysis makes lactate continuously

As described by editor Schurr in the introductory chapter, the conclusion that glycolysis makes lactate is a central issue; by definition, glycolysis makes lactate (Schurr, 2014). Whether glycolysis produces acid, or not, is a different, and perhaps unresolved issue. That glycolysis produces lactate normally, under fully aerobic conditions is perhaps best exemplified by results of studies on resting muscle (Stanley et al., 1986; Richardson et al., 1998; Bergman et al., 1999b), the healthy beating heart (Gertz et al., 1981, 1988; Bergman et al., 2009), the normally functioning brain (van Hall et al., 2009; Wyss et al., 2011; Glenn et al., 2015), and in other diverse tissues following enteral carbohydrate nutrition (Foster, 1984; Meyer et al., 2002). That lactate production is greatly increased during muscular exercise can be readily observed (Stanley et al., 1985; Mazzeo et al., 1986; Bergman et al., 1999b; Messonnier et al., 2013), but again lactate production in working muscle during graded exercise up to maximum is not due to oxygen insufficiency (Richardson et al., 1998), and for that matter, the elevation in circulating lactate concentration during prolonged exercise cannot be attributed solely to net lactate release from working muscle (Ahlborg and Felig, 1982; Brooks et al., 1998). Rather, some other large tissue bed, perhaps the integument (Johnson and Fusaro, 1972) is responsible for elevated blood lactate concentration and turnover rates when there is no net lactate release from working muscle (Ahlborg and Felig, 1982; Brooks et al., 1998).

Importance of the fact that lactate can be produced in particular cellular compartments and from those sites can enter the interstitium and vasculature from where it can reach adjacent or anatomically distributed cells, organs, and tissues to affect important functions was recognized in 1984 (Brooks, 1984). At that time data from several sources were evaluated in light of original data on glucose and lactate fluxes in resting and exercising, untrained and endurance trained laboratory rats (Brooks and Donovan, 1983; Donovan and Brooks, 1983). The exchange of lactate among cellular sites of production and removal was termed the “Lactate Shuttle” (Brooks, 1984).

In humans and other mammals the extent of lactate production is typically overlooked because removal (disposal) rate balances production (appearance) rate. In other words, with typical concentration measurement technology low and stable blood lactate levels belie high turnover (production and removal) rates. And, even when arterial-venous [a-v] concentration differences and tissue blood flow measurements are available, in the absence of isotope tracer technology turnover rate is unknown because of rapid turnover within the tissue bed of interest. Restated, basing conclusions about the dynamics of lactate or any other metabolite solely on concentration data is intellectually equivalent to taking the US census in the morning and evening of the same day, and then upon seeing no significant change in population numbers concluding that nothing happened in the population during that day. Happily, many entered the population, and regrettably also, some exited. Hence, concentration data provide an incomplete view of lactate turnover (flux) except in conditions of sudden and large changes when homeostatic imbalances in the relationship between production and removal are indicated.

**Figure 1:**
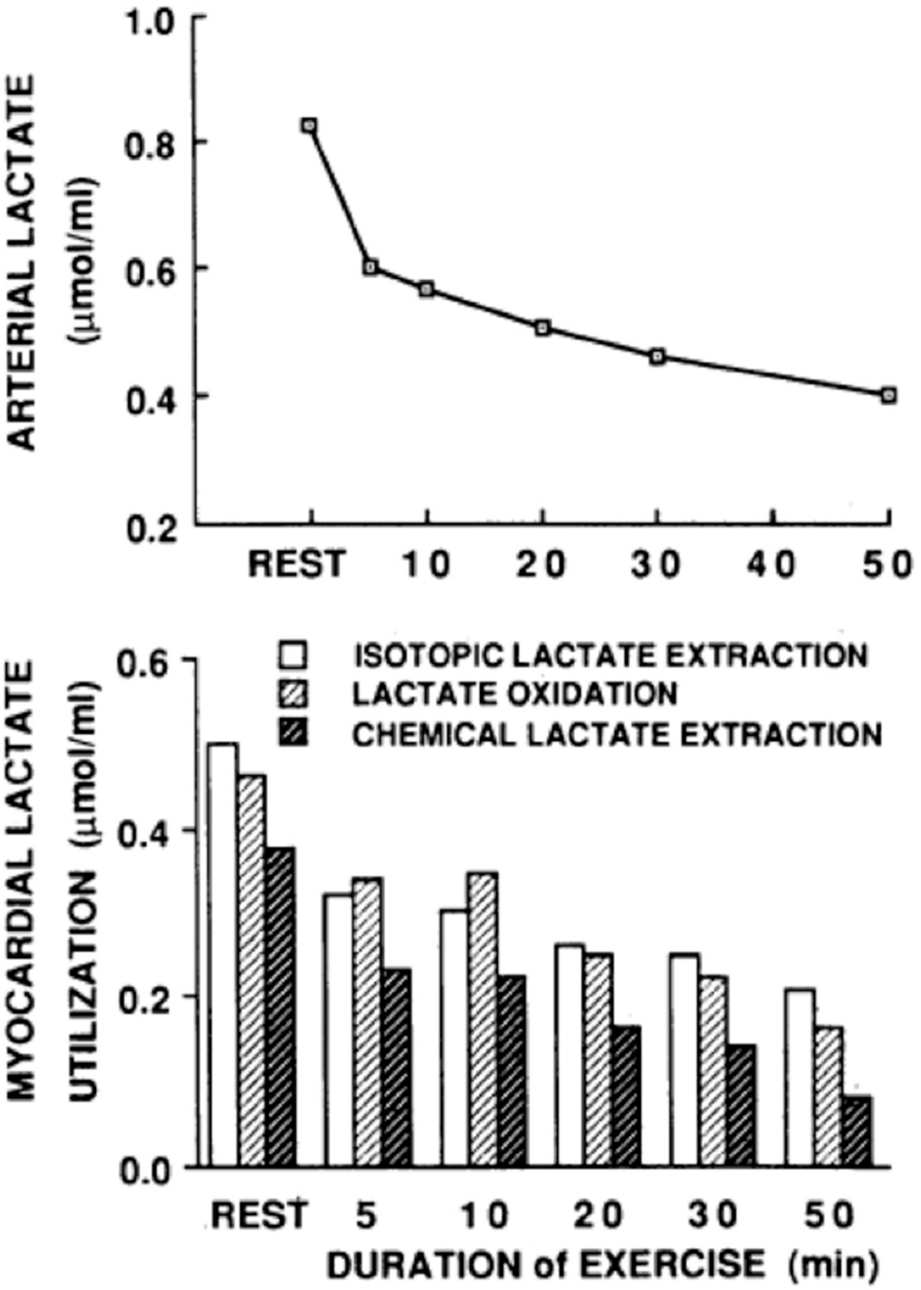
**Arterial lactate level (upper graph) and a comparison of myocardial tracer-measured (isotopic) lactate extraction, lactate oxidation, and chemical extraction based on (a-v) and myocardial blood flow (lower graph) are shown for a trained bicyclist whose arterial [lactate] fell during moderate-intensity exercise**. The results show that net lactate exchange measurements underestimate total lactate production, that most lactate produced is oxidized, and that lactate uptake is concentration dependent. From Gertz et al. (1988).

In resting or working muscle (Stanley et al., 1986; Bergman et al., 1999b), the heart in normal sinus rhythm at rest or under load (Gertz et al., 1981, 1988; Bergman et al., 2009), and in other tissues such as skin (Johnson and Fusaro, 1972), lactate production and net release are the norm. Restated, idling or working skeletal muscles and heart simultaneously produce and consume lactate (Stanley et al., 1986; Bergman et al., 1999b) as shown by means of simultaneous tissue blood flow arterial-venous difference ([a-v], [a-v]Δ, or AVD) measures for lactate concentration and isotopic enrichment (Stanley et al., 1986). Depending on conditions such as nutritive state, power output, duration of activity, and arterial lactate concentration, working muscle may switch from net release to consumption (Brooks et al., 1998; Bergman et al., 1999b). As well, tissues other than working muscle can produce lactate, and if arterial lactate concentration rises sufficiently, working muscle can become a lactate consumer as seen in men exercising at sea level, on acute exposure to 4300 m altitude, and after 3 weeks of acclimatization to 4300 m altitude on Pikes Peak (Brooks et al., 1998). For the heart, net uptake is typical (Gertz et al., 1988), with few reports of lactate net release from the working heart (Gertz et al., 1981). In the example shown (Figure 1), net lactate release from the legs of a trained bicyclist causes arterial lactate concentration to rise at exercise onset. However, as exercise continues, lactate clearance mechanisms, including lactate uptake and oxidation by the heart reduce the circulating lactate load. Data in Figure 1 also show that myocardial lactate uptake and oxidation are concentration dependent. In the figure “chemical lactate” extraction is equal to “net uptake” based on [a-v] for chemical lactate concentration and myocardial blood flow, which is equivalent to the cerebral metabolic rate of lactate (CMRlac) terms used in studies of cerebral lactate metabolism employing measurements of [a-v] and cerebral blood flow (CBF). The dependence of cellular lactate exchange on concentration difference is due to the fact that cell membrane lactate transport proteins (Garcia et al., 1994, 1995) are symports that co-transport lactate anions and hydrogen ions (Roth and Brooks, 1990a,b). Typically then, cells release lactate because of intracellular glycolysis and lactate accumulation, whereas lactate consumer cells such as heart, liver, kidneys, and red skeletal muscle contain a high mitochondrial density which lowers intracellular [lactate] relative to arterial and interstitial [lactate]. In the brain, as in other tissues lactate exchange among neurons and other cell types depends not only on the metabolic rates within cells, but also on the extracellular environment which is influenced by cellular metabolism as well as vascular delivery and removal.

Given the existing literature on lactate metabolism in human muscle and heart it should not be surprising that the healthy human brain displays similar features (Glenn et al., 2003, 2015; van Hall et al., 2009; Wyss et al., 2011), brain lactate turnover is the norm and occurs during net release as well as net uptake. In brain, as well as other tissues, glucose is taken up, glycolysis occurs, and lactate is produced. However, because lactate is also taken up, the CMR for lactate depends on the balance of cerebral production plus uptake vs. disposal, which is mainly oxidation (Glenn et al., 2015).

**Figure 2:**
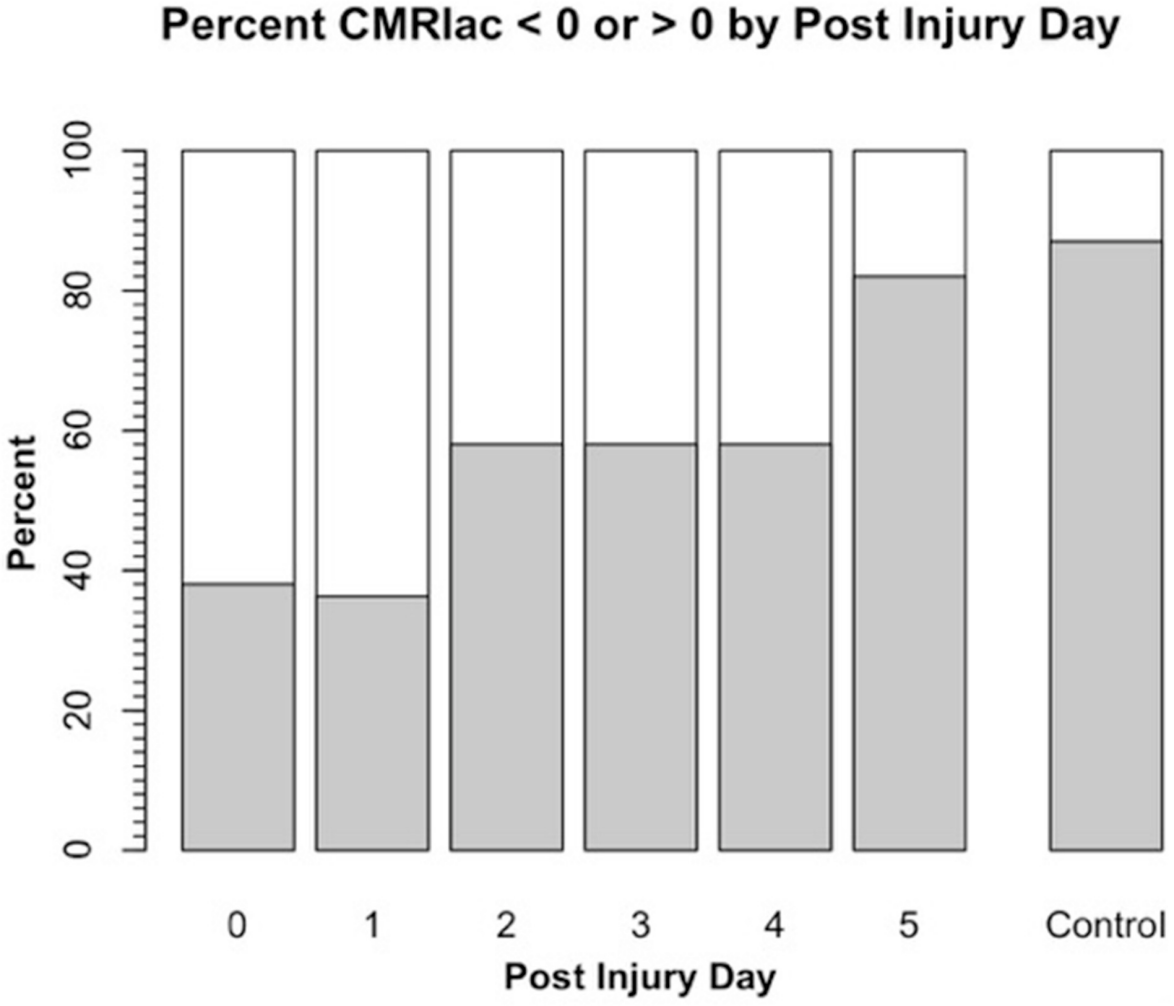
**Cerebral metabolic rate (CMR) for chemical lactate [CMRlac = (CBF) (a-v)lac] over time in patients with severe TBI**. To illustrate the change in CMRlac over time, data presented as percentage of patients demonstrating net cerebral lactate uptake (i.e., CMRlac < 0, white area) compared percentage of patients demonstrating cerebral net lactate release (i.e., CMRlac > 0, dark area). Patients display wide variability and significant changes over time with regression to control values of net cerebral lactate release over time. As illustrated in Figure 1, CMRlac underestimates total lactate production. Redrawn from Glenn et al. (2003) and ongoing studies with control values courtesy of T. C. Glenn.

Science is difficult enough, and in the case of lactate metabolism understanding is complicated by the use of different terms in different fields. Hence, we need to define terms as they appear in this review. For brain, cerebral metabolic rate for glucose (CMRgluc) equals the arterial-venous difference for glucose (i.e., [a-v]gluc) times CBF: CMRgluc = (AVDgluc) (CBF). Similarly, for lactate (CMRlac) equals the arterial-venous difference ([a-v]lac) times CBF: CMRlac = (AVDlac) (CBF). As already noted, in skeletal (Stanley et al., 1986; Bergman et al., 1999b) and cardiac muscle (Gertz et al., 1988; Bergman et al., 2009), and lung (Johnson et al., 2012) physiology, the term net lactate exchange is equivalent to CMRlac and in these instances the negative sign (−) indicates net lactate release, whereas a positive sign (+) indicates net lactate uptake. For Figure 2 on cerebral net lactate exchange (>0 sign, and dark area) indicates net cerebral lactate release, whereas the (<0) sign and light area indicates lactate uptake as shown for control subjects and patients after suffering traumatic brain injury (TBI). In the first days following injury most patients display net cerebral lactate uptake transitioning to net release as in controls after several days of intensive care. This said, whether positive or negative, the CMR for lactate underestimates production because of simultaneous production and removal within brain tissue (van Hall et al., 2009; Glenn et al., 2015) as it does in other tissues such as skeletal muscle (Stanley et al., 1986; Bergman et al., 1999b) and heart (Gertz et al., 1988).

As illustrated by the use of equivalent terms “net glucose uptake” and “CMRgluc” in muscle and brain metabolism fields, a larger hurdle must be negotiated to achieve understanding of the significance of tissue turnover. For tissues such as muscle and brain that are net glucose consumers, tracer- and chemical measures of glucose yield equivalent results because glucose is not produced in muscle (Bergman et al., 1999a) or brain (Glenn et al., 2015). However, muscle (Stanley et al., 1986; Bergman et al., 1999b), and brain (van Hall et al., 2009; Glenn et al., 2015) simultaneously consume and produce lactate. Therefore, to know muscle or brain lactate turnover rate both uptake and release need to be measured and summed to calculate total lactate production.

Realization that tissue “net lactate” and “CMRlac” provide data on only part of the story leads to insight into meaning of the term “lactate production,” and its use, or misuse as too often investigators will mistake the terms lactate “accumulation” and “net release” to mean “lactate production.” Such misuse of terminology is more than careless; the misuse reflects lack of understanding and can lead to misinterpretation of data and missed opportunities for intervention in the intensive care unit (ICU).

## Glycolysis and “hyperglycolysis” after cerebral injury

Glucose uptake and glycolysis are features of normal cerebral metabolism, but trauma affects CMRgluc and glycolysis in specific ways. In particular, there is a relative increase in glycolysis, a “hyper-glycolysis” that is a recognized feature of cerebral metabolism immediately after injury (Bergsneider et al., 1997). As part of the sympathetically mediated fight and flight mechanism (Selye, 1950; Selye and Fortier, 1950), both glycogen and glucose are precursors to glycolytic flux. However, compared to muscle in which glycogen content is typically 1.25 g/100 g (1 mMol/100 g tissue) (Hultman, 1967; Krssak et al., 2000), and liver in which glycogen storage can be several times greater than in muscles of well-nourished individuals (20–40 mMol/100 g tissue) (Nilsson and Hultman, 1974; Roden et al., 2001), cerebral glycogen content is very small (0.3–0.4 μMol/100 g tissue) (Oz et al., 2007). Accordingly, it is understandable that cerebral glycogen is rapidly recruited and depleted following acute injury. Limited cerebral glycogen reserves point to the importance of the continuous delivery of carbohydrate energy sources to the brain, always (Glenn et al., 2003; van Hall et al., 2009), and especially following injury (Glenn et al., 2003; Vespa et al., 2012). Not to confuse cerebral substrate delivery and use with the US Federal Budget, the human brain is definitely “pay as you go,” with deficits in oxygen, substrate, both or either, having significant effects on tissue function and viability.

In the field of cerebral metabolism, the term “hyperglycolysis” is a relative term used to connote the extent of metabolic crisis following injury. Following injury, depression of brain functions are indicated by the decrements in cerebral oxygen consumption rate (CMRO_2_) as well as glucose uptake and use (CMRgluc). Hence, the presence of hyperglycolysis is indicated by a fall in the normal molar ratio (MR), or “Metabolic Ratio” of 6/1 for CMRO_2_/CMRgluc, the stoichiometry for complete glucose oxidation being given as: 6 O_2_ + C_6_H_12_O_2_ → 6 CO_2_ + 6 H_2_O. The fall in cerebral MR following injury is indicative of a relative increase in the role of glycolysis leading to cerebral lactate production (Glenn et al., 2015), as well as an increase in pentose phosphate pathway activity (Dusick et al., 2007). Reiterated, the state of “hyperglycolysis” indicates an increase in glycolysis relative to cerebral oxygen consumption.

Because brain cells depend on glycolysis from carbohydrate energy sources, while at the same time possessing limited capacity for glycogen reserves, the importance of cerebral glucose delivery in health and disease cannot be overstated. The homeostatic setpoint for blood glucose concentration is 5 mM (≈100 mg%), whereas typical circulating blood [lactate] is <1 mM (<9 mg%), and [pyruvate] and [β-OH butyrate] are in the μM range (<1 mg%). Restated, vascular glucose mass is 10-fold or more greater than the circulating alternative CHO energy sources combined. Hence, it is to be expected that maintaining euglycemia from exogenous nutritional support is of major importance in the neural ICU. This can perhaps be illustrated best by the better outcomes of TBI patients as a result of careful assessment and early enteral and parenteral nutritional support (Wang et al., 2013).

However, without knowledge of the metabolic and nutritional status of the individual patient, the practice of medicine using means-based administration of nutritional support and insulin therapy (van den Berghe et al., 2001) can result in over- and underfeeding, hyper- and hypoglycemia (Van den Berghe et al., 2006; Henderson and Finfer, 2009; Marik, 2009; Myburgh and Chittock, 2009) because assessments of the patient are based solely on blood metabolite concentration measurements. The use of [glucose] and the treatment of hyper- and hypoglycemia with insulin without consideration of individual flux can potentially result in medical care that creates the condition that is then subsequently treated. Infusion of exogenous glucose at a rate greater than patients' individual glucose turnover will cause hyperglycemia. This is then treated using insulin to maintain glycemia. A better approach would be to utilize the autoregulatory functionality that is intact (Glenn et al., 2014) and monitor glucose production rate (i.e., Ra) to control the individual patient's [glucose]. Given current and anticipated technological advances, such as stable isotope technology and rapid analysis, it should be possible for attending clinicians to continually monitor an individual patient's body energy state (BES) and to deliver precision nutritive care by adjusting enteral and parenteral nutrient delivery over the course of treatment. Restated, while the field of nutrition and pathophysiological metabolic pathways is pursued heavily by scientists and physicians worldwide, and late vs. early enteral and parenteral guidelines are desired for the treatment of the critically ill and injured (Casaer et al., 2011b), means based titrating blood [glucose] with insulin (Meijering et al., 2006) or standardized equations describing caloric needs for healthy individuals (Harris and Benedict, 1918) cannot describe the intra-patient metabolic and nutritional variability (Glenn et al., 2014).

## Hints at elevated cerebral lactate metabolism post-injury

Brain injuries are complicated and produce highly variable effects, not only between patients, but temporally within patients with seemingly confounding and contradictory effects. For example, hyperglycolysis is typical post-TBI (Bergsneider et al., 1997), and was observed by Glenn et al., who saw TBI to significantly decrease cerebral MR (Glenn et al., 2003). However, even though injury depressed cerebral MR, many TBI patients demonstrated cerebral net lactate uptake in the hours post injury (Figure 2). Hence, puzzlingly hyperglycolysis coincided with a positive CMRlac, or increased net lactate uptake. UCLA investigators took the apparently paradoxical results to indicate that something extraordinary was happening to brain CHO metabolism post injury (Glenn et al., 2003).

That the human brain could take up and use lactate as a fuel, not simply take up and accumulate lactate was entirely consistent with extensive literature reports on cerebral metabolism in laboratory rodents and rodent brain slices and tissue preparations. Not only do astrocytes and neurons contain lactate transporters (MCTs) necessary for cellular uptake (Pellerin et al., 2005; Pierre and Pellerin, 2005), but importantly also mitochondria of neurons contain MCTs and other components of the mitochondrial lactate oxidation complex (mLOC) (Hashimoto et al., 2008) necessary for neuronal lactate oxidation. Again, as described in the introductory chapter (Schurr, 2014), in comparison to glucose, rodent brain slices and neuronal preparations preferentially take up and oxidize lactate in comparison to glucose (Schurr, 2006, 2008; Schurr and Payne, 2007). Although controversial a decade ago (Glenn et al., 2003), the hint of cerebral lactate oxidation following injury has been elaborated upon with results of basic science studies on laboratory animals (Schurr and Payne, 2007) and clinical research studies on humans (Smith et al., 2003; Gallagher et al., 2009; Ichai et al., 2009, 2013; Wyss et al., 2011; Jalloh et al., 2013; Bouzat et al., 2014; Glenn et al., 2014).

## Body supports the brain

**Figure 3:**
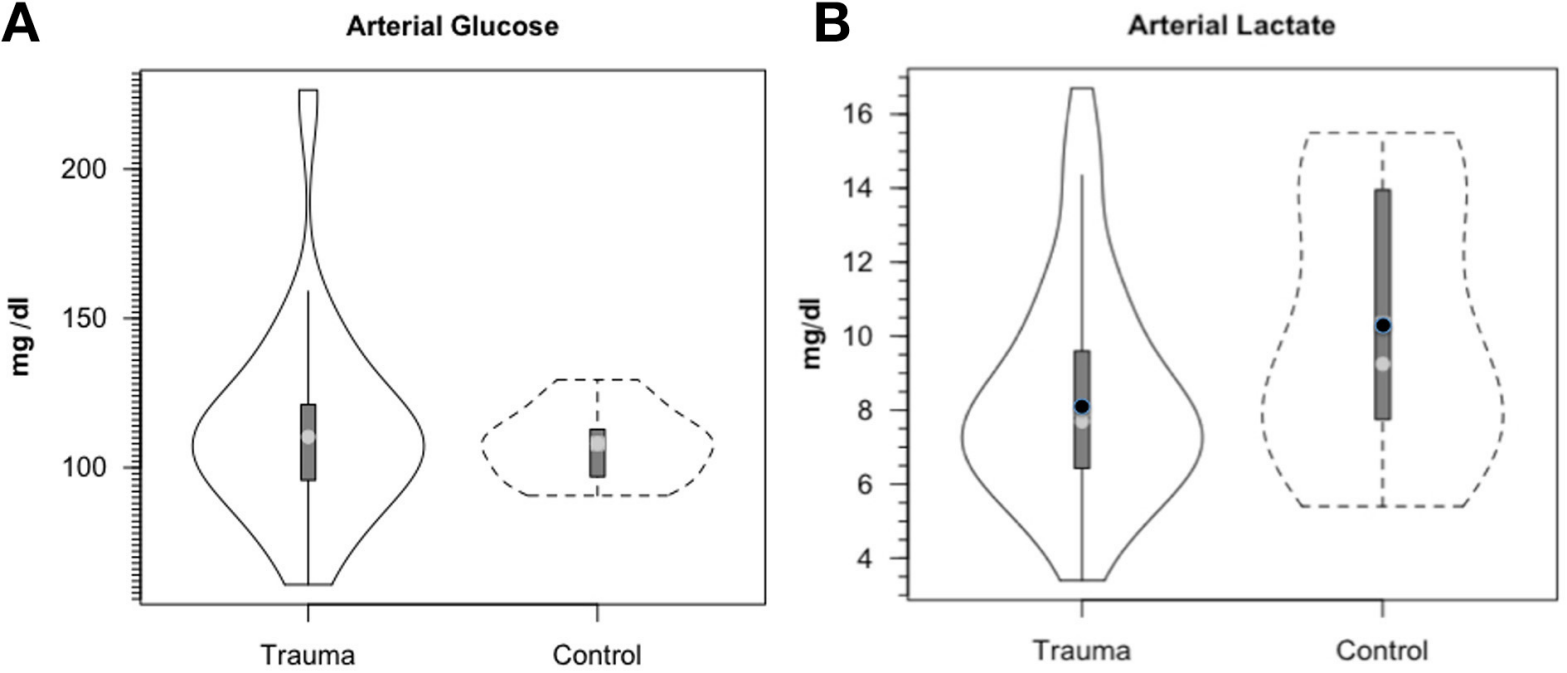
**Arterial glucose (A) and lactate concentrations (B) in control and TBI patients**. Values were constant over time, so mean values for min 60, 90, and 120 min are shown. Solid lines represent TBI patients (*n* = 12) while dashed lines are normal control subjects (*n* = 6). This and subsequent figures depict the following components: median (light circle), mean (dark circle), standard deviation (heavy vertical bar), box-plot whisker (thin vertical bar) and a kernel density estimation of the data distribution (replacing the box-plot's rectangular depiction) following Hintze and Nelson (1998) as visualized by R package “Caroline” (Schruth, 2012). When coincident, the median circle symbol obscures the mean symbol. TBI patients demonstrated arterial glucose and lactate levels similar to those in healthy control subjects. From Glenn et al. (2014).

**Figure 4:**
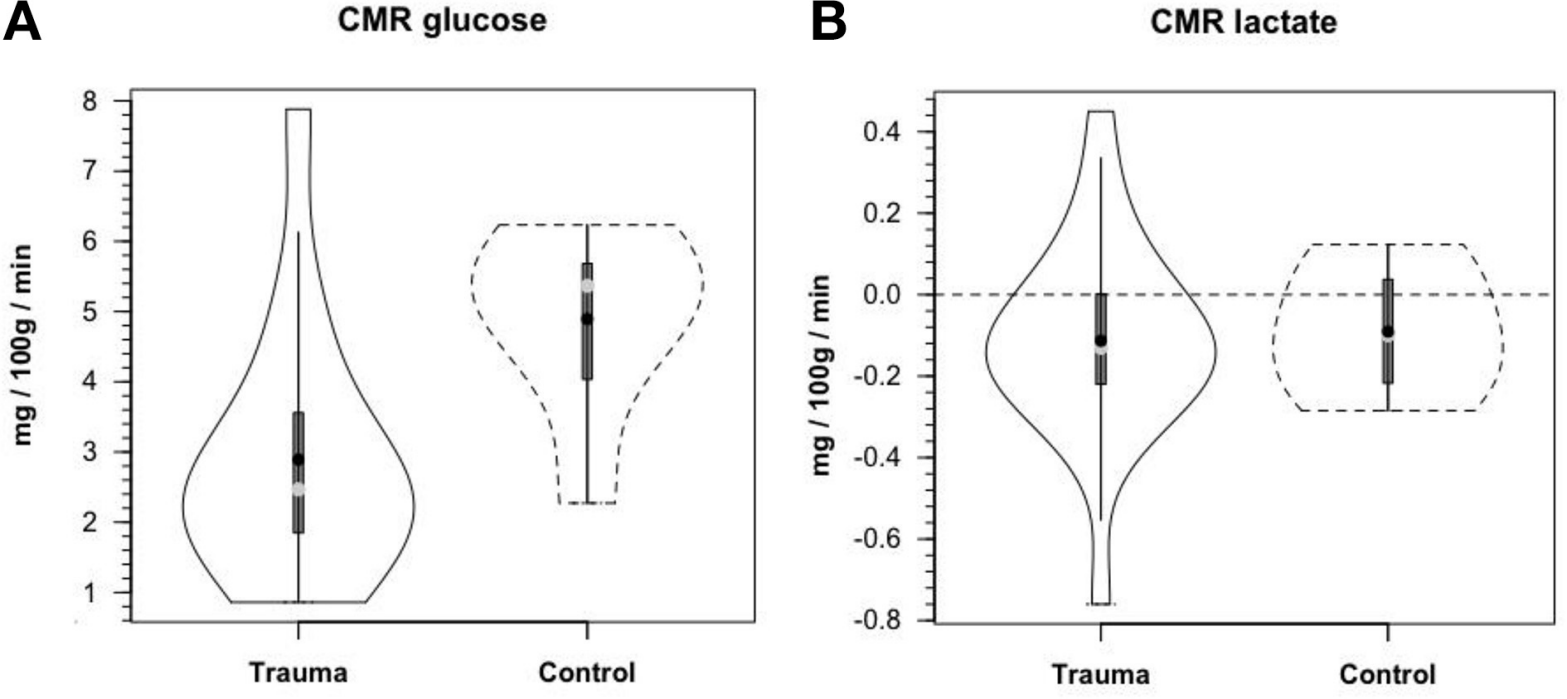
**Cerebral metabolic rates (CMR) for glucose (A) and lactate (B), in control and TBI patients; CMR = (Metabolite AVD) (Cerebral Blood Flow), alternatively termed net exchange**. Values were constant over time, so mean values for min 60, 90, and 120 min are shown. Solid lines represent TBI patients (*n* = 12) while dashed lines are normal control subjects (*n*–6); other aspects of the figure described in the legend to Figure 3, above. Figures show net glucose uptake and net lactate release. Compared to values in controls, CMRgluc, but not CMRlac, was depressed in TBI patients. From Glenn et al. (2015).

Along the way to studying cerebral glucose-lactate interactions in patients following TBI an unexpected result emerged on our TBI patients given standard of care treatment in state of the art facilities by the dedicated and highly trained health care professionals. This was the herculean effort of the body to mobilize energy resources for the injured brain (Glenn et al., 2014). With reference Figure 2, patients were enrolled in our isotope tracer studies involving use of primed-continuous infusions of [6,6-^2^H_2_]glucose (i.e., D2-glucose) and [3-^13^C]lactate to track whole-body and cerebral glucose-lactate interactions in TBI. Patients were studied as soon as permission of legal representatives could be obtained, usually 96–140 h after injury at which time numerous parameters were approaching control levels. The time lag had to do with mechanics of securing permission of patients' legal representatives to conduct studies on patients receiving standard of care treatment. Consequently, at the time of study arterial glucose (Figure 3A) and lactate (Figure 3B) concentrations in patients were not significantly different from those in fasting control subjects. However, compared to values in control subjects, in TBI patients CMRgluc was significantly depressed (Figure 4A) (*p* < 0.01), whereas consistent with Figure 2 values, CMRlac was similar to control values (Figure 4B).

**Figure 5:**
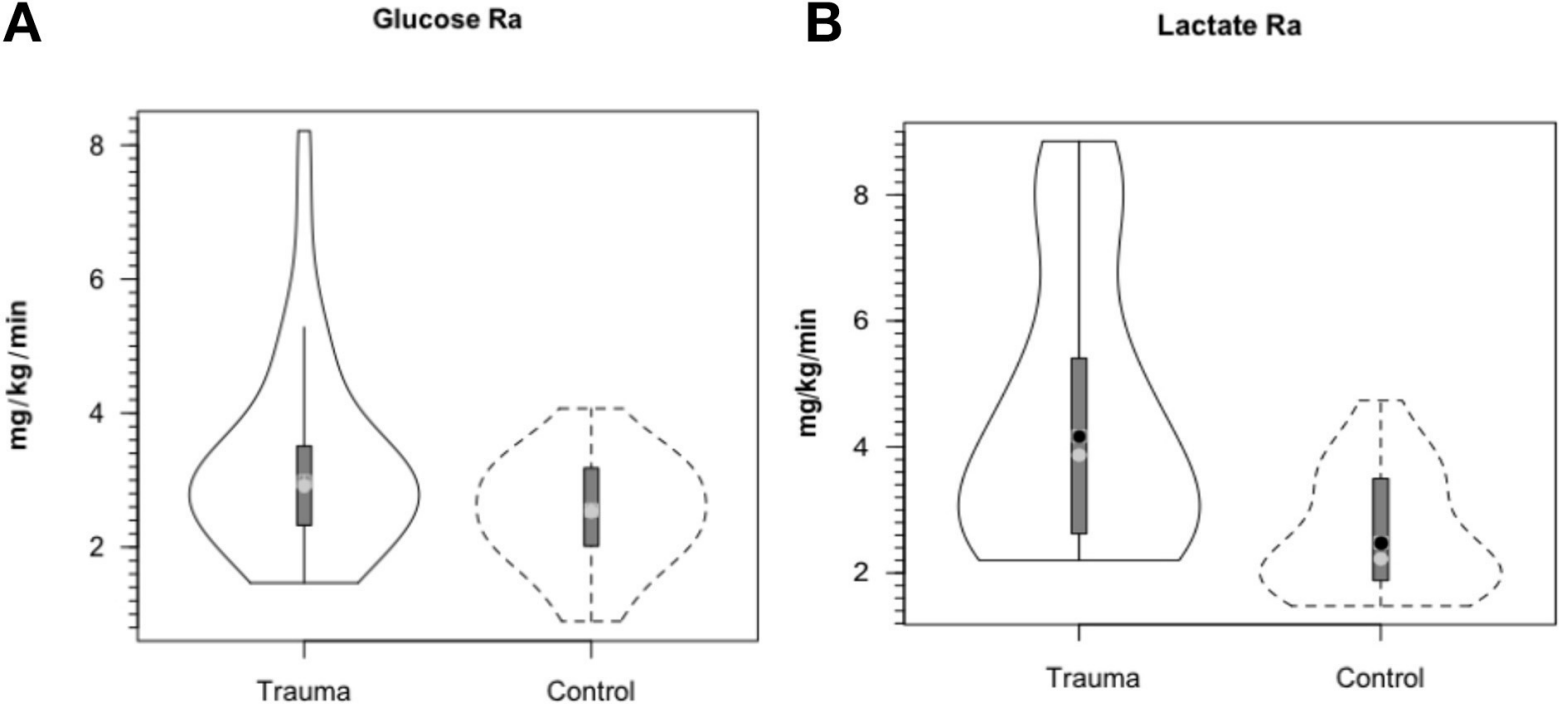
**Hepatic (+ renal) glucose production, Ra (A) and body lactate appearance rate (B) in control and TBI patients**. Values were constant over time, so mean values for min 60, 90, and 120 min are shown. Solid lines represent TBI patients while dashed lines are normal control subjects. Due to variability in TBI patients, the tendency for higher glucose production was NSD (*P* = 0.06). However, whole-body production was ≈ 90% higher in TBI patients than healthy controls (*P* < 0.01). From (Glenn et al., 2014).

As with glucose and lactate concentration levels, whole body glucose turnover (Ra shown) in treated TBI patients was similar to values in controls (Figure 5A). Due to variability in TBI patients, the slightly higher glucose Ra in patients was not significantly different (*P* = 0.06), but trends toward indicating a hypermetabolic state, or, in comparison to exercising controls, a moderate intensity of exercise similar to a brisk walk. Again the intra-patient variability demonstrated that some patients were more hypermetabolic than others, and therefore would require increased nutritional support as their body energy stores are depleted more rapidly. However, even though arterial [lactate] was similar in control subjects and patients, blood lactate turnover (Ra shown) was elevated by ≈90% in TBI patients compared to controls (Figure 5B). Because in this investigation we lacked AVD and blood flow measurements across tissues other than brain, we can only speculate that the sites of lactate release were muscle (Bergman et al., 1999b) and the integument (Johnson and Fusaro, 1972) as is found in normal physiology. Since blood glucose and lactate concentrations were similar in controls and patients the natural tendency was to conclude that not much was happening other than blood glucose homeostasis had been achieved. However, such a conclusion would not have been correct.

**Figure 6:**
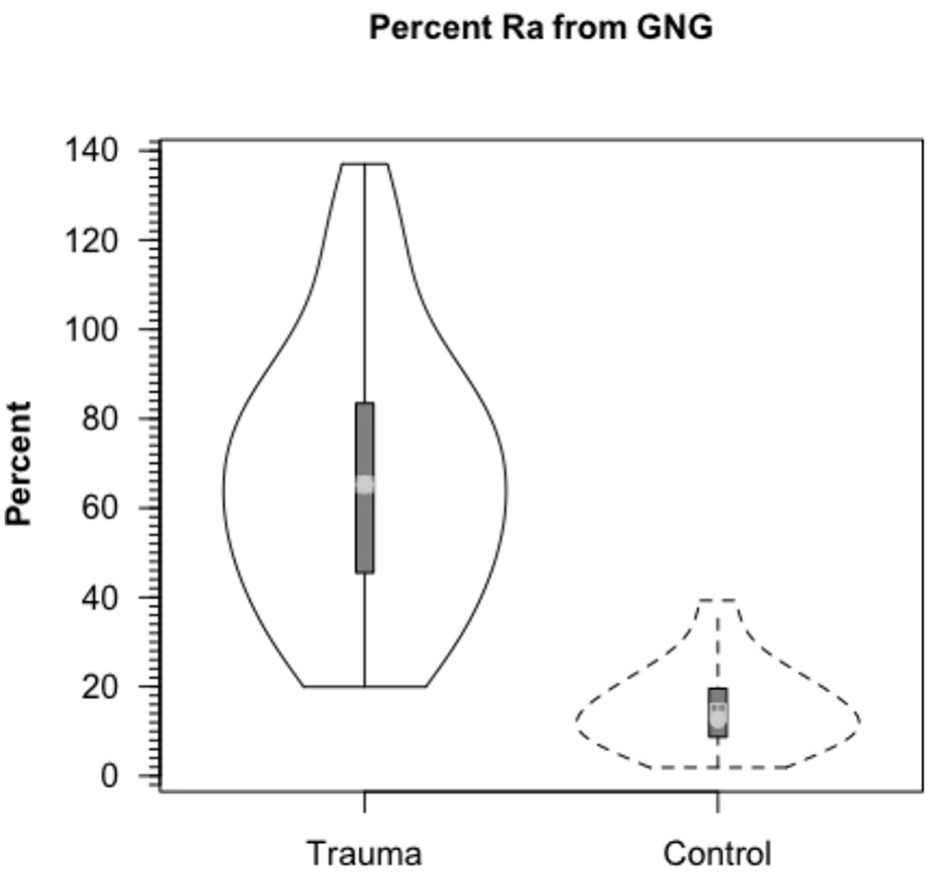
**Percent contribution of lactate to glucose production (gluconeogenesis, GNG) in healthy controls (dashed lines, *n* = 6) and TBI patients (solid lines, *n* = 12)**. Values significantly greater following TBI, *p* < 0.05. Trauma caused a major change in gluconeogenesis from lactate. From Glenn et al. (2014).

Because we could measure the incorporation of ^13^C into blood glucose from infused [3-^13^C]lactate, the rate of gluconeogenesis (GNG) from lactate and the percentage of circulating glucose formed from lactate could be measured. Compared to values of 15.2% of glucose Ra from lactate in control subjects, most (67.1%) circulating glucose in TBI patients came from lactate (Figure 6). These dramatic, and fundamentally paradigm changing results made possible because of the use of dual isotope tracer studies would not have been envisioned from contemporary measures of blood metabolite concentrations (Figures 3A,B). With the linked-goals of reducing time in the intensive care unit and improving patient outcomes, and recognition that TBI patients given best standard of care treatment are in a catabolic state, presents challenges, but offers opportunities to improve TBI patient care and improve patient outcomes by providing nutritive support to the injured, but recovering brain.

## Brain runs on lactate both directly and indirectly

The central role of glucose in sustaining brain metabolism has long been recognized (Scheinberg and Stead, 1949; Scheinberg et al., 1965; Cahill et al., 1968; Sokoloff, 1973; Dienel and Hertz, 2001), with classic and contemporary results (Glenn et al., 2003, 2015) in accord. These rare, but extremely important data contribute in major ways to the understanding of cerebral metabolism in normal and pathological conditions, including the formation of dietary reference intakes for carbohydrate nutrition (Medicine, 2005), the values for Estimated Daily Requirement (EAR) and Recommended dietary Allowance (RDA = EAR ± 2SD) for carbohydrate to provide cerebral needs 100 and 130 g/day, respectively.

Contemporary measurements show that despite hyperglycolysis on injury, CMRgluc is decreased in the days following TBI (Figure 4A) (Glenn et al., 2015). Again, in the patients studied, chemical concentration based measurement of CMRlac was not significantly different from control values (Figure 4B) (*P* > 0.05). Like decreased CMRO_2_, decreased CMRgluc following TBI may reflect the presence of intrinsic cerebral mechanisms to allow the injured brain to “rest” in recovery. Alternatively, decreased cerebral metabolism following injury may be representative of an injury-induced metabolic crisis, the latter being associated with poor patient outcomes (Stein et al., 2012). Hence, the emphasis in this review is to optimize substrate availability to promote healing of the injured brain.

**Figure 7:**
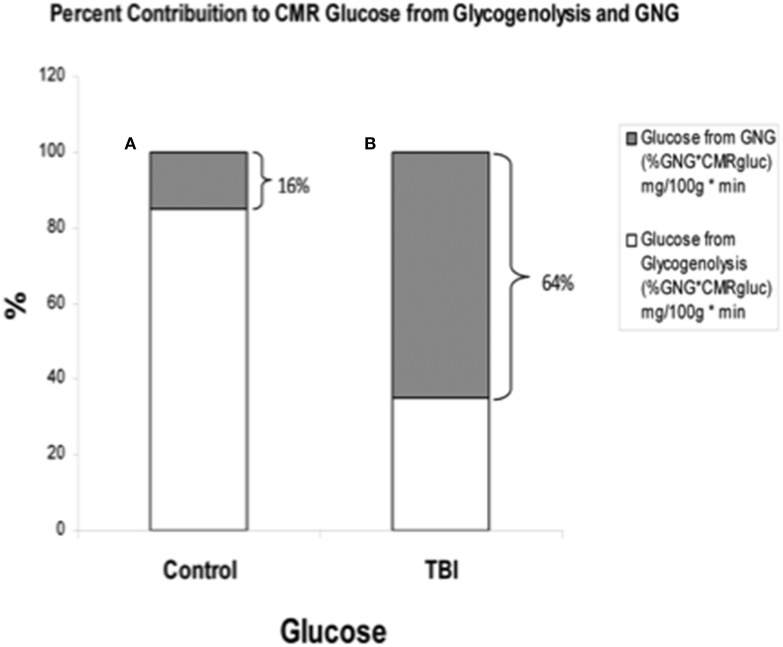
**By supporting gluconeogenesis, lactate indirectly supports CMR glucose**. The relative contributions to CMR glucose from hepatic gluconeogenesis (GNG), and glucose from hepatic glycogenolysis in healthy control subjects (Left, **A**), and TBI patients (Right, **B**). A comparison of **(A)** (control) and **(B)** (TBI) shows the large increase in percentage cerebral glucose uptake contributed by GNG from lactate following TBI. From (Glenn et al., 2015).

By comparison with results from studies on healthy control subjects the importance of GNG from lactate in supplying glucose for the brain is shown in Figure 7. In TBI patients the pattern of glucose production from hepatic gluconeogenesis (GNG) vs. glycogenolysis (GLY) is very different from that in healthy, postabsorptive control subjects in whom GNG contributes only 16% of hepatic glucose Ra whereas GNG contributes 64% to glucose Ra in TBI. Clearly, following TBI endogenously produced lactate definitively supports brain metabolism via GNG; we term this “indirect” brain fueling following TBI (Glenn et al., 2015). However, lactate is also directly taken up and oxidized by injured and healthy brains; we term this “direct” brain fueling by lactate.

Until advent of advanced technologies, such as the use of isotope tracers (van Hall et al., 2009; Glenn et al., 2015), cerebral lactate metabolism in healthy and brain-injured individuals was anticipated from results of AVDlac and CMRlac measurements (Figures 2, 4B) and experimentation on animal brain preparations (Schurr, 2008), but the extent of cerebral lactate turnover could not be assessed because of simultaneous lactate production and oxidative disposal within the tissue.

**Figure 8:**
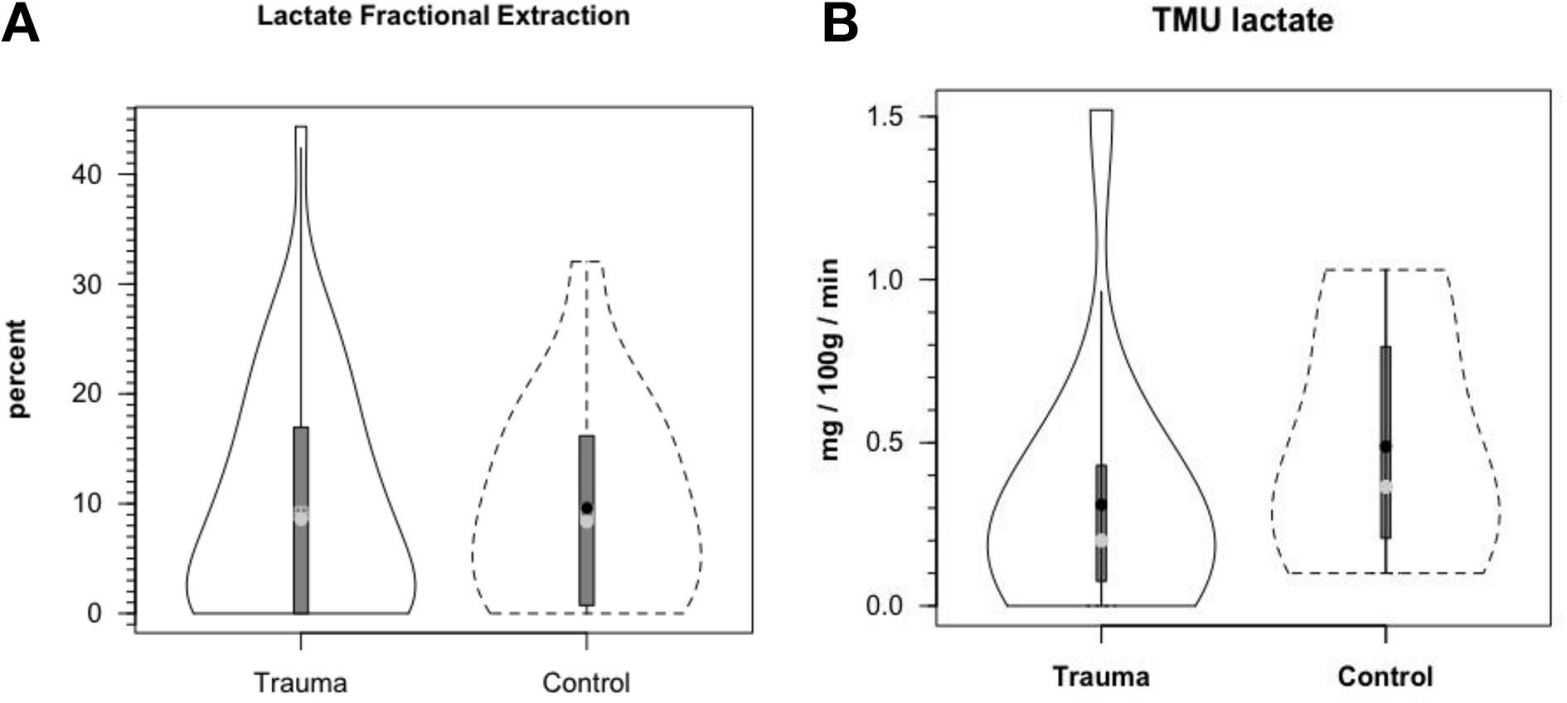
**Cerebral lactate fractional extraction (A) and tracer-measured cerebral lactate uptake (B) in healthy control subjects and TBI patients**. Solid lines represent TBI patients while dashed lines are normal control subjects. Both fractional extraction and lactate uptake are preserved in TBI patients indicating plausibility of increasing cerebral lactate uptake by raising arterial [lactate] by means of exogenous lactate infusion. From (Glenn et al., 2015).

**Figure 9:**
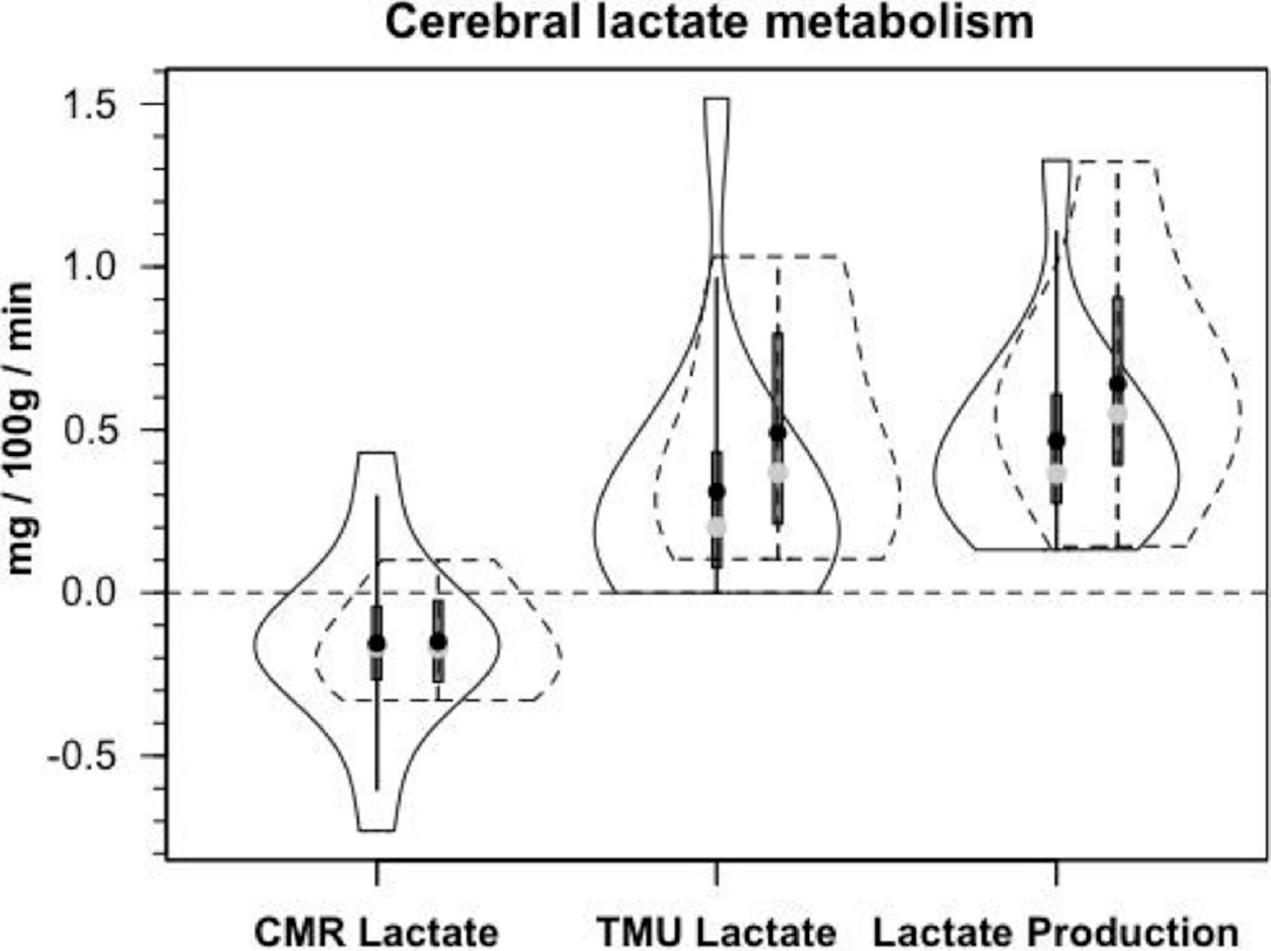
**Components of cerebral lactate metabolism: Release (CMRlac), Tracer-Measured Uptake and Total Cerebral Lactate Production = TMU-CMRlac**. Solid lines represent TBI patients while dashed lines are normal control subjects. The use of [^13^-13C]lactate tracer allow a very different view of cerebral lact production. Should CMRlac be taken as a measure of lactate production, total lactate production would be grossly underestimated. Regardless, whether estimated from CMR or total lactate production, control subjects and TBI patients show similar capacities for lactate uptake, release and production. From Glenn et al. (2015).

Figure 8A shows cerebral lactate fractional exaction (FExlac) to approximate 10% in both healthy control subjects and those suffering TBI (Glenn et al., 2015). Incidentally, the FEx for glucose also approximates 10% in healthy subjects and TBI patients (Glenn et al., 2015), so the value of 10% FEx for lactate is in the range of biological plausibility. Nonetheless, knowing CBF, arterial [lactate] and FExlac, cerebral tracer-measured lactate uptake (TMUlac) can be determined and compared to ^13^CO_2_ excretion. With our moderate and severe TBI patients studied 5.7 ± 2.2 days after injury tracer-measured cerebral lactate uptake was not different from values measured in healthy control subjects (Figure 8B). Then, knowing and summing net lactate release (CMRlac) and cerebral tracer-measured lactate uptake (TMUlac), total cerebral lactate production can be determined (Figure 9). As shown in Figure 9, the conceptual model of cerebral lactate metabolism developed from seeing concentration-based depictions, such as arterial [glucose] and [lactate] (Figure 3), and CMRgluc and CMRlac (Figure 4), is very different from that developed from knowledge of total cerebral lactate production (Figure 9). To reiterate, new information of whole-body and cerebral glucose-lactate interactions show that glucose metabolism is suppressed following TBI, but lactate metabolism is intact. This knowledge provides impetus to explore the possibility of supporting cerebral carbohydrate metabolism and improving patient outcomes following injury by providing formulations containing lactate and other monocarboxylates.

**Figure 10:**
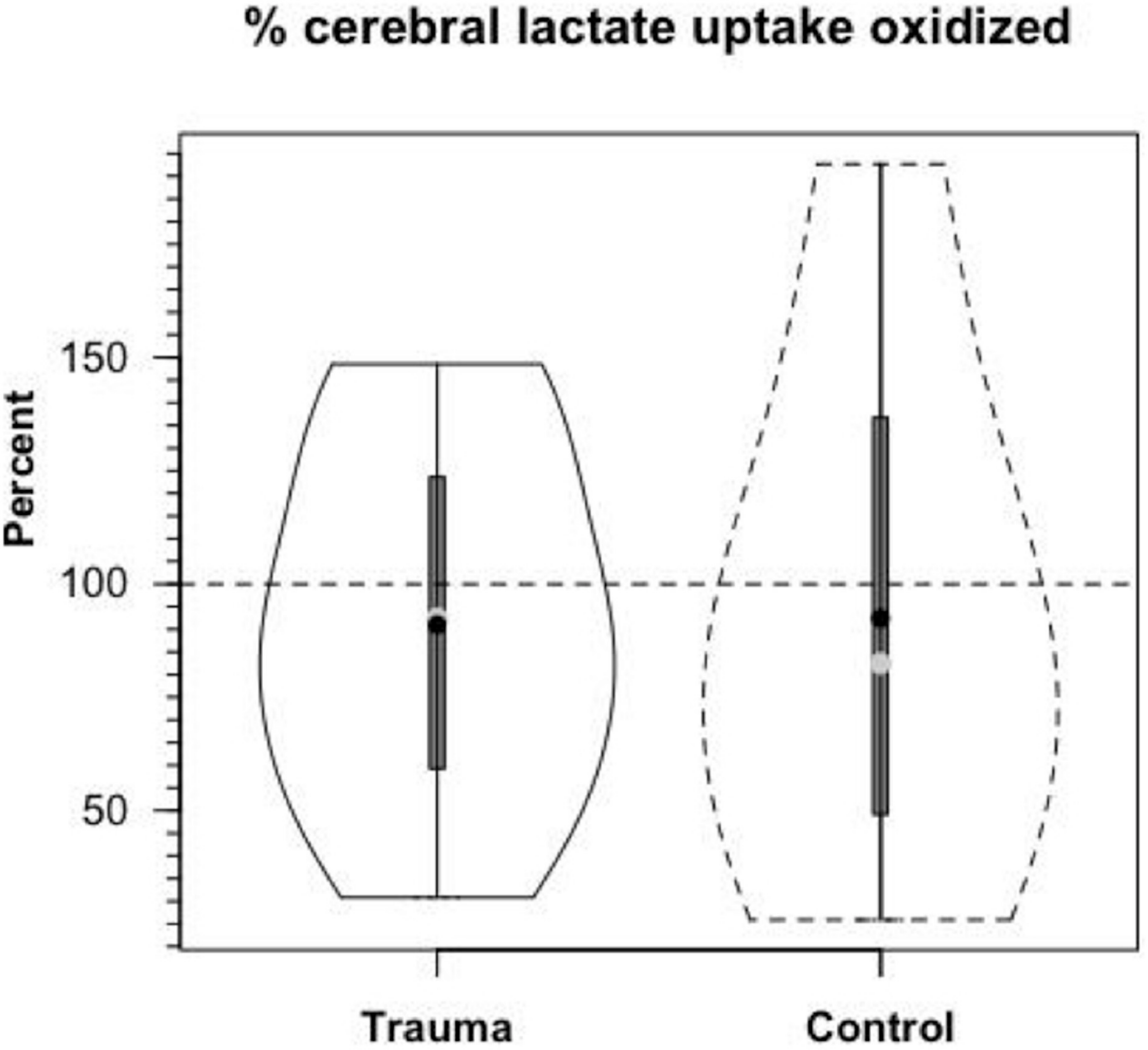
**Cerebral lactate Oxidation in control and TBI patients**. Values given in relative terms (% lactate uptake that is oxidized). Lactate taken up by healthy controls and TBI patients is oxidized directly within the tissue. Values corrected for the contribution to cerebral release of ^13^CO_2_ from the oxidation of ^13^C-glucose produced from circulating [3-^13^C]lactate. From (Glenn et al., 2015).

**Figure 11:**
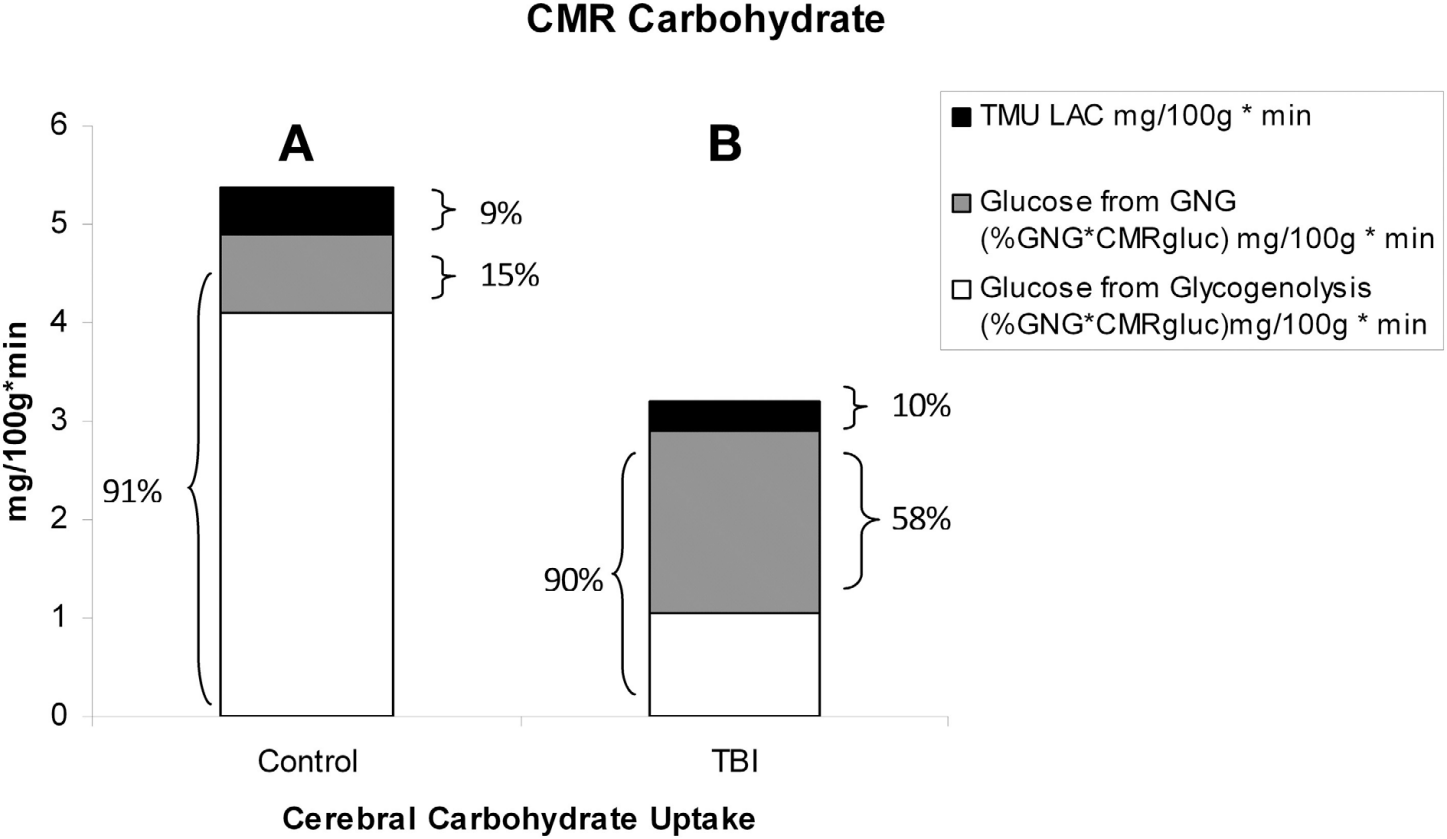
**Absolute and relative contributions to total cerebral carbohydrate (CHO) from lactate, glucose from gluconeogenesis (GNG), and glucose from hepatic glycogenolysis (GLY = Glucose Ra – GNGgluc) in healthy control subjects (Left, A), and TBI patients (Right, B)**. Compared to **(A)**, panel **(B)** shows the decrease in total CHO use and CMRgluc following TBI, but increased contributions of lactate and glucose from GNG to total cerebral CHO uptake after TBI. A comparison of **(A)** (control) and **(B)** (TBI) shows the large increase in percentage cerebral CHO uptake contributed by lactate, directly, or indirectly, in comparison to healthy controls who have hepatic GLY available for to support CMRgluc. From Glenn et al. (2015).

Based on measurements of cerebral tracer-measured lactate uptake (TMU) (Figure 8B) and simultaneously measured ^13^CO_2_ excretion into the jugular bulb, cerebral lactate oxidation is essentially 100% (Figure 10). Among various things the data indicate that lactate entering the brains of healthy controls and TBI patients is not simply stored, but utilized as a fuel energy source. Importantly, the results (Figure 11) show that the brain oxidizes lactate directly, which is supported by evidence of the mLOC being present in the only species (rat) examined to date (Hashimoto et al., 2008).

With the benefit of simultaneous CBF as well as arterial and jugular bulb concentration and isotope enrichment measurements for glucose and lactate it has been possible to partition total cerebral carbohydrate uptake in healthy controls and TBI patients (Figure 11). These results are consistent with what has been known about cerebral metabolism, but importantly they also provide novel insights into cerebral carbohydrate metabolism in healthy control subjects and TBI patients as the sum of glucose and lactate uptake equals total cerebral carbohydrate metabolism.

In healthy controls cerebral glucose uptake accounts for 15–25% of blood glucose disposal (Scheinberg and Stead, 1949; Scheinberg et al., 1965; Cahill et al., 1968; Sokoloff, 1973; Dienel and Hertz, 2001), and for the first time this has been validated using tracer technology (Glenn et al., 2015). The percentage varies depending on conditions, with time since eating a major factor because GNG is suppressed by the arrival of dietary energy, CHO and macronutrients (Trimmer et al., 2002). Now, because of our simultaneous measurements of body and brain glucose and lactate fluxes we gain additional insights into total cerebral carbohydrate metabolism, and therefore introduce the term CMRCHO. In well-nourished postabsorptive controls glucose production from hepatic glycogen provides 76% of cerebral carbohydrate needs, with 15% of the total of glucose produced from lactate via gluconeogenesis, the latter representing the indirect contribution of lactate. Direct cerebral lactate uptake represented 9% of cerebral CHO needs in control subjects (Figure 11A). In contrast, while CMRgluc is suppressed following brain injury, and glucose still represents 90% of CMRCHO, gluconeogenesis from lactate provides 58% CHO energy supply, and the relative contribution of lactate uptake remains at 10% (Figure 11B). Hence, most energy supplied to the injured brain comes directly, or indirectly from lactate.

Like Figures 4A, 6 shows that CMRgluc is suppressed in TBI patients. However, besides the effect of TBI on cerebral CHO needs and CMRgluc, the side-by-side comparison in Figure 7 shows a very different pattern of cerebral carbohydrate energy source in patients following TBI. While glucose still provided most CHO-energy, most (59%) glucose came indirectly from lactate via GNG, and 9% came from direct lactate uptake in TBI patients making the total lactate or lactate-derived contribution 68% of total CHO energy as opposed to 25% in controls.

## Clinical applications: exogenous lactate infusion studies

### Brain needs

As described here and in related papers (Glenn et al., 2014, 2015), several recent realizations lead to the question: “is vascularly infused lactate an alternative brain fuel?” On one hand, if lactate is the major oxidative energy source, how can it be considered as an alternative when it is, in fact “the fuel?” However, on the other hand, if cerebral glycolysis is blocked after injury, then lactate formulations are substances that health care givers can administer to supplement, or augment vascular nutrient delivery to an injured brain. In the latter case, formulations containing lactate salts, esters and other compounds would effectively augment the typical dextrose solutions currently used to support TBI patients. At a minimum, the exogenous glucose should not be administered at a rate greater than the glucose turnover rate of the individual patient. Such a practice would inevitably result in hyperglycemia and require insulin treatment. Seemingly, a better alternative would be to support glycemia by providing lactate and other monocarboxylate formulations that would allow the liver and kidneys to maintain euglycemia as is typically seen with exogenous lactate infusions (Miller et al., 2002a,b; Bouzat et al., 2014).

Although not widely recognized, exogenous vascular infusion of sodium lactate-lactic acid mixtures has been utilized to study substrate kinetics in dogs (Issekutz and Miller, 1962), rats (Guo et al., 2009; Johnson et al., 2011, 2012), and humans (Miller et al., 2002a,b, 2005; Ichai et al., 2009, 2013). The latter studies established short-term safety of the method of infusing concentrated lactate solutions for several hours into healthy men with good renal function to manage the sodium load and respiratory alkalosis (Miller et al., 2005). Importantly, studies have commenced with the intent of determining the effects of sodium lactate infusion in cerebral metabolism following TBI (Ichai et al., 2009; Bouzat et al., 2014; Glenn et al., 2015). In addition to the infusion of sodium lactate, investigators have experimented with infusion of hypertonic sodium pyruvate into swine to evaluate viability of that approach for cardiac resuscitation and neuroprotection following cardiac arrest (Sharma et al., 2005, 2008; Ryou et al., 2009, 2010, 2012). In control beagles, nominal arterial lactate and pyruvate concentrations are similar to those in humans (1.0 and 0.1 mM, respectively), but, as used, sodium pyruvate infusion increases arterial pyruvate and lactate concentrations to 3.5 and 8 mM, respectively (Sharma et al., 2005), and so pyruvate infusion may be an effective tool in managing blood and tissue [L]/[P] while raising arterial [L] and [P].

### Lactate and its alternatives for brain fueling

Above we discussed some of the issues related to fueling the injured brain, either by providing glucose or an alternative such as lactate or keto acids such as pyruvate, acetoacetate or β-hydroxybutyrate. Aside from what glycolysis produces and neurons respire, numerous factors need consideration, some of which are described here.

Lactate: We have advanced the idea that lactate enters the mitochondrial reticulum via one or several MCT isoforms and interacts with the mitochondrial Krebs Cycle and respiratory apparatus via a mLOC in muscle (Brooks et al., 1999a,b; Dubouchaud et al., 2000; Hashimoto et al., 2006) and brain (Hashimoto et al., 2008), and others have advanced the idea that pyruvate enters the mitochondrial matrix via pyruvate carrier isoforms (Bricker et al., 2012; Herzig et al., 2012). However, all seem to agree that the monocarboxylates lactate and pyruvate traverse cell membranes via MCT1 or MCT4 (Brooks, 2009; Divakaruni et al., 2013). Plasma membrane MCT isoforms have different affinities for monocarboxylates (Roth and Brooks, 1990a,b), but it is clear also that in blood the monocarboxylates appear in concentrations that differ by an order of magnitude or more. For instance, the nominal blood lactate/pyruvate ratio ([L]/[P] or L/P) in arterial blood of < 10 can rise to > 300 in venous effluent of working human muscle (Henderson et al., 2007). Related to the L/P in blood are the effects of LDH in erythrocytes and the lung parenchyma that raise the L/P during each circulatory passage (Johnson et al., 2011, 2012).

Operating under the assumption that because it is the product of glycolysis and biological brain fuel (Schurr, 2014), we (Miller et al., 2002a,b; Glenn et al., 2014) and others (Oddo et al., 2012; Ichai et al., 2013; Bouzat et al., 2014) experimented on supplementing nutritive supply to the injured brain with formulations containing lactate or lactate anion. As described above, lactate supports glycemia without causing hyperglycemia because of hepatic autoregulation of blood [glucose]. Further, lactate is taken up directly and oxidized by the healthy as well as injured brain, and, importantly, in the ranges studied, uptake is not saturation limited (Figure 8A). Importantly, and in contrast to the case for working skeletal muscle in which lactate supplementation substitutes for glucose, following TBI in which glycolysis is partially blocked, lactate supplementation via a 2 mM lactate clamp did not decrease CMRgluc, but instead raised total cerebral CHO uptake (unpublished data). This so called “sparing” of blood glucose can then potentially be used for other cerebral metabolic priorities of glucose such as the neuroprotective pentose phosphate pathway (Bartnik et al., 2005; Dusick et al., 2007).

Short, several hour infusions of sodium lactate appear to result in manageable sodium and alkalotic stresses in healthy controls (Miller et al., 2005) and TBI patients with good renal and respiratory function (Bouzat et al., 2014). Likely, however, for longer durations infusions of other lactate formulations including organic salts (arginyl lactate), esters (glycerol tri-lactate or N-acetyl lactate) or other compounds will need to be developed and tested for safety and effectiveness in human subjects.

Pyruvate: To reiterate from above, Mallet and colleagues have developed sodium pyruvate infusion procedures and have done extensive testing in animal models (Sharma et al., 2008; Ryou et al., 2012). Typically the investigators utilize 2 M sodium pyruvate solutions for infusion via central venous lines (Sharma et al., 2008). However, sodium pyruvate solutions are unstable and give rise to noxious degradation products. To our knowledge, neither sodium pyruvate nor pyruvate derivatives such as ethyl pyruvate and N-acetyl pyruvate have been tested for safety and effectiveness in human subjects. Moreover, the biological rationale for infusing pyruvate is questionable because it is rapidly converted to lactate in blood (Sharma et al., 2005; Johnson et al., 2011, 2012) and so the question arises what is the effective moiety taken up by healthy and injured tissues.

Another advantage of exogenous lactate over pyruvate supplementation relates to another chronic problem in the management of TBI patients, that is the complication of hyperglycemia with dextrose infusion and poor control of glycemia (Vespa et al., 2012). A potential advantage of infusing lactate, a gluconeogenic precursor, as opposed to dextrose for maintaining glycemic control is to take advantage of hepatic autoregulation of glucose Ra. At present, extant data indicates that in both healthy individuals (Miller et al., 2002a,b) and TBI patients exogenous lactate infusion does not result in hyperglycemia (Bouzat et al., 2014). In contrast, sodium pyruvate infusion results in hyperglycemia in beagles (Sharma et al., 2005).

Given that both lactate and pyruvate are likely to traverse plasma membrane, and hence blood-brain barrier thresholds by MCTs that are capable of transporting either monocarboxylate, a logical suggestion is to consider infusing mixtures of both lactate and pyruvate, particularly if it is found efficacious to affect the circulating L/P. For the present, however, given the liabilities of infusing pyruvate compounds, there seems to be no practical advantage of infusing pyruvate over lactate. In this context some would argue that pyruvate is an effective ROS scavenger and is neuro- and cardio protective (Mallet and Sun, 2003; Sharma et al., 2008), but similar claims can be made for lactate (Rice et al., 2002; Holloway et al., 2007; Schurr and Gozal, 2011; Alessandri et al., 2012; Herzog et al., 2013; Bouzat et al., 2014).

Ketones: The “ketones” acetoacetate and β-hydroxybutyrate cross plasma membrane and blood-brain barriers via facilitated, MCT-mediated transport (Roth and Brooks, 1990a,b), but the specialized MCT isoform appears to be MCT2 (Prins and Giza, 2006). Because acetoacetate and β-hydroxybutyrate gain access to neurons via a unique isoform, thus minimizing crosstalk and competition for transport among MCT isoforms, the approach of using ketogenic diets to support cerebral healing in pediatric populations is an area of active research (Prins and Hovda, 2009; Prins, 2012). Therefore, by extension of ongoing efforts it is entirely reasonable to consider augmenting neuroplegic solutions for iv infusion in the treatment of acute TBI with acetoacetate and β-hydroxybutyrate.

As with other candidates for inclusion in neuroplegic solutions acetoacetate and β-hydroxybutyrate would have advantages and disadvantages. The ketones would contribute to whole body and cerebral substrate availability, and thus would represent direct fuel energy sources for the body and brain. However, ketones would be unlikely to contribute to the maintenance of glycemia via GNG. As well, acetoacetate and β-hydroxybutyrate would by necessity be delivered as sodium salts with efficacy dependent on good renal function.

### Body and brain in competition for nutrients in the ICU [body energy resource but nutrient sink]

Despite being treated according to current standard of care protocols, in state of the art facilities, by talented and dedicated health care professionals, as indicated by the very high rate of gluconeogenesis (Figure 5), our patients were undernourished. In our companion reports (Glenn et al., 2014, 2015) we describe herculean efforts of the body, liver and kidneys to maintain glycemia via GNG. Above we described possible uses of neuroplegic formulations to sustain the injured brain and promote healing, but efficacy of supporting the injured brain by providing intravenous support needs to be considered within the context of overall, whole-body nutrition. Whether delivered via a central or peripheral line, parenteral nutrition will go to where the circulation takes it. Relative to other tissues, the brain is hypermetabolic and is relatively well perfused in healthy and severely brain injured individuals. However, most enteral and parenteral nutrition will go elsewhere. For example, infused lactate is removed mainly by oxidation in skeletal muscle (Bergman et al., 1999b; Miller et al., 2002a,b; Emhoff et al., 2013). Hence, despite valiant efforts to support the injured brain, both directly and indirectly from endogenous sources (Glenn et al., 2014, 2015), so far as parenteral nutrition is concerned the body is in competition with the brain for resources. Hence, it is to be anticipated that the intravenous infusion of neuroplegic solutions following TBI need to be considered on the background of overall enteral nutrition so that overall nutrient and energy availability is sufficient to meet needs of the brain and other tissues whether they be healthy or injured.

Assessing nutritional adequacy of patients in the ICU is a technical challenge. At best, results of indirect calorimetry are difficult to interpret and the technology is difficult to employ, especially in head injury cases, or when a patient is artificially ventilated. Standardized equations (Harris and Benedict, 1918) force regression to population means, but cannot be expected to precisely meet individual patient needs. The use of nitrogen balance, albumin and prealbumin measurements are also difficult to employ and there are significant time lags between measurements and ongoing changes in nitrogen balance. No wonder the literature is rich with controversy over the benefits, or liabilities of providing enteral or parenteral nutrition to patients (McClave et al., 1998; Petros and Engelmann, 2006; Griffiths, 2007; Loh and Griffiths, 2009; Casaer et al., 2011a). However Wang et al. (2013) show in a meta-analysis review of TBI treatments that providing enteral and parenteral nutritive support early result in better patient outcomes (Wang et al., 2013). In our view, such uncertainty involves an inability to deliver personalized, precision care to patients. Hence, new methods of assessing BES in the ICU need to be found to aid clinicians in delivering energy and nutrients sufficient to cover needs for the body and brain. In this regard, %GNG is an excellent candidate biomarker for assessing BES in the ICU, but technology needs to be developed and validated for clinical use.

### The confused state of understanding on lactate clearance

Results of our investigations indicate that caution needs to be applied when using the term “lactate clearance” as a biomarker of the severity of traumatic injury (Zhang and Xu, 2014). Without the use of isotope tracers, clearance is calculated as net metabolite change over time with lactatemia and lactic acidosis harbingers of poor outcome. In our investigation we infused [3–13C]lactate tracer to determine metabolic clearance rate (MCR = Rd/[lactate]a), units being (ml/kg/min). In our investigation lactate production (Ra) and disposal (Rd) rates were significantly elevated following TBI (Figure 4B) (Glenn et al., 2014), while arterial lactate concentration ([lactate]a) was the same in control subjects and TBI patients (Figure 3B). This means that lactate MCR was very high and significantly greater, not lower, in TBI patients compared to controls. Additional tracer studies will be required to establish the relationship between lactate MCR and outcome following TBI.

## Summary

Trauma to the brain results in a metabolic crisis (Stein et al., 2012; Vespa et al., 2012), or crises as the mechanism of injury may involve trauma to other body parts. The injured person is typically hypermetabolic even if cerebral metabolism is suppressed following injury (Glenn et al., 2003). Together, our results (Glenn et al., 2014, 2015) and those of others (Gallagher et al., 2009; van Hall et al., 2009; Jalloh et al., 2013; Bouzat et al., 2014) indicate a significant role for lactate in cerebral normo- and pathophysiology. Given that CMRgluc is suppressed following cerebral injury, it's possible to take advantage of the Lactate Shuttle mechanism of supplying energy to bypass the restriction in glycolytic flux and spare limited glucose reserves for other functions such as pentose phosphate pathway activity.

As reviewed above, sodium lactate infusion is a logical first step in translating new knowledge of cerebral lactate metabolism into procedures and practices to provide nutritive support to the injured brain. However, other lactate compounds or formulations containing additional or other amendments may offer still greater potential to nourish the injured brain in the context of an injured or malnourished body. In the space available we have shown new and dramatic results on body-brain interactions in moderate and severe TBI patients. Those results not only show direct and indirect nutritive support of the body for the brain, but the results should alert scientists and clinicians alike to the possibility that attempts to provide parenteral, vascular, support for the brain will be met by an eagerly needy, and far larger body corpus that can easily outcompete the injured brain for glucose and monocarboxylate alternatives. Therefore, by using glucose production rate and %GNG as biomarkers for BES in individual patients health care providers would have actionable data upon which to personalize individual patient needs for delivery of energy and nutrients required to support both brain and body needs. In practical terms, with knowledge of BES health care professionals would have knowledge to provide energy and macronutrients to meet corporal needs, as indicated by %GNG in the range of 25% (Horning and Brooks, 2012a,b). Then, via parenteral routes monocarboxylate formulations designed specifically to supplement cerebral energy needs could be delivered in an effort to support cerebral repair and recovery following injury (Horning and Brooks, 2012a,b).

Today the field of precision medicine focuses predominately on genomics, but all variations of the omics, (i.e., application of panomic analysis to individual disease) will participate (NIH, 2011) in efforts to utilize the concept of precision medicine to improve health care delivery, particularly in the area of managing trauma. In fact, other omics constituting precision medicine (e.g., proteomics, metabolomics, and now fluxomics), as illustrated through the use of %GNG as a biomarker for BES and treatment using formulations targeting injured tissue, already might be areas of interest. Indeed, segments of the biotech, medtech, digital health, and life sciences industries are spearheading medical applications and exploring opportunities. With basis of knowing an individual patient's BES, health care providers will be able to practice precision medicine through use of personalized prescriptions to deliver exogenous enteral and parenteral nutritive formulations to maintain a body energy state, that neither over- or underfeeds patients, but, importantly, provides directed nutrient support for the injured brain. A personalized approach to precision body energy state will therefore enable the clinicians to support both the body and the brain during recovery from trauma.

### Conflict of interest statement

The authors declare that the research was conducted in the absence of any commercial or financial relationships that could be construed as a potential conflict of interest.
